# Clinical Profile and Treatment Outcomes of Malignant Mixed Mullerian Tumors of the Uterus: A Single-Center Experience

**DOI:** 10.7759/cureus.48079

**Published:** 2023-10-31

**Authors:** Niranjan Vijayaraghavan, Dinesh Ravikumar

**Affiliations:** 1 Medical Oncology, Apollo Hospitals, Chennai, IND; 2 Medical Oncology, Madras Medical College, Chennai, IND

**Keywords:** adjuvant therapy, prognostic factors, recurrence, radiation, chemotherapy, malignant mixed mullerian tumor

## Abstract

Background

A malignant mixed Mullerian tumor (MMMT), otherwise known as carcinosarcoma, is a rare and aggressive malignancy involving the uterus. Despite aggressive treatment, they have poor outcomes. We performed this study to analyze the clinical profile, prognostic features, and treatment outcomes of patients treated for MMMT of the uterine corpus in our center.

Methodology

A study sample from a database of patients treated at a tertiary care center in South India between January 2015 and December 2019 was analyzed retrospectively. A total of 29 non-metastatic patients were included in the study. The diagnosis of MMMT was confirmed by a pre-operative biopsy specimen or endometrial curettage. Information regarding the patient, tumor characteristics, details of the surgery, adjuvant therapy, and follow-up details were collected retrospectively. Disease-free survival (DFS) and overall survival (OS) were plotted using the Kaplan-Meier method, and the log-rank test was used to identify any significant prognostic factor. Statistical analyses were performed using IBM SPSS software, version 21 (IBM Corp., Armonk, NY).

Results

The population’s median age was 60 years (interquartile range (IQR): 52-65). The homologous type was the most common pathology seen in 21 patients. The median size of the tumor was 7 centimeters (cm) (IQR: 6-10). Around two-thirds of the patients received adjuvant radiation therapy. Paclitaxel and carboplatin-based adjuvant chemotherapy were used in 60% of the patients. Nearly 90% of the recurrences were distant recurrences. During the follow-up, 17 patients developed a recurrence. The median DFS was 12 months (95% CI: 7.7-16.2). The median OS was 26 months (95% CI: 10.6-41.3). The three-year OS rate was 42%. Patients with an age >60 years had a median overall survival of 11 months, compared to 37 months for patients <60 years (p = 0.026). The median overall survival of patients with tumor sizes >10 cm was 12.5 months, and it was 35 months when the tumor size was less than 10 cm (p = 0.03). Patients receiving radiotherapy (RT) had an improved survival (39 months) compared to those who did not receive RT (12.5 months) (p = 0.046). The median overall survival for patients after the recurrence was 12 months. As most patients were elderly with an Eastern Cooperative Oncology Group (ECOG) performance status of two at baseline, only a few patients were fit for second-line chemotherapy.

Conclusion

A malignant mixed Mullerian tumor is an aggressive tumor of the uterine corpus with poor survival rates. Surgical resection remains the mainstay of treatment. Adjuvant radiation therapy improves survival. The age of the patient, myometrium invasion, tumor size, and positive peritoneal cytology were found to be prognostic factors.

## Introduction

A malignant mixed Mullerian tumor (MMMT), also known as carcinosarcoma, is a rare malignancy commonly seen in the uterine corpus. Other sites include the cervix, ovary, and peritoneum. They are biphasic tumors exhibiting carcinomatous and sarcomatous components [1]. Because of its rarity, there is no consensus or randomized trial to decide on the right treatment [2]. Surgery remains the mainstay of treatment. Adjuvant therapies like radiation and chemotherapy have been tried to improve survival. Despite all these efforts, the five-year overall survival (OS) rate is between 33% and 39% [3]. We performed this retrospective study to identify the prognostic factors that affect the survival of patients with MMMT of the uterine corpus.

This article was previously presented as a meeting abstract at the European Society For Medical Oncology (ESMO) Gynaecological Cancers Congress, Valencia, Spain, on June 17th, 2022.

## Materials and methods

This retrospective study sample was drawn from a database of patients treated at Madras Medical College, a tertiary care center in Chennai, South India, between January 2015 and December 2019. Patients who presented with recurrent disease to our institute after treatment for primary disease at another hospital were excluded from the analysis. A total of 36 patients with MMMT from our database were identified. We excluded four patients who had upfront metastatic disease and three patients with incomplete medical records. Patients were classified according to the 2009 International Federation of Gynecology and Obstetrics (FIGO) staging for endometrial carcinoma. In our study, we included a total of 29 patients with clinical stages I-III MMMT of the uterine corpus with no clinical evidence of distant metastasis at presentation. We included two patients who were found to have distant metastasis during laparotomy and received curative treatment. The diagnosis of MMMT was confirmed by a pre-operative biopsy specimen or endometrial curettage. Patients who were eligible for surgery underwent total abdominal hysterectomy with bilateral salpingo-oophorectomy, bilateral pelvic lymph node, and para-aortic lymph node dissection and omental sampling. Patients who had a good Eastern Cooperative Oncology Group (ECOG) performance status of 0 to 2 received adjuvant chemotherapy and radiotherapy (RT). Pelvic external beam radiotherapy (EBRT) was delivered using a megavoltage beam on a linear accelerator with a 2D technique to cover the vaginal vault and bilateral pelvic lymph nodes. A dose of 50.4 Gy in 28 fractions was delivered with five fractions per week. Subsequently, four cycles of adjuvant chemotherapy with paclitaxel (175 mg/m2) and carboplatin (area under the free carboplatin plasma concentration versus time curve (AUC 5)) were administered. The patients were routinely followed up by clinical and radiological examinations every three months after therapy. Information regarding the demographic profile, clinical features, tumor characteristics, details of the surgery, adjuvant therapy, and follow-up were collected retrospectively.

Descriptive statistics were summarized using percentages and frequencies. Continuous variables were summarized using the mean with standard deviation or median with interquartile range. Twenty-nine patients were included to analyze disease-free survival, defined as the time from diagnosis to recurrence of the disease or death. All patients were included to analyze OS, defined as the time from diagnosis to death from any cause. The Kaplan-Meier method was used to plot the survival curves, and the log-rank test was used to identify any significant prognostic factor. Statistical analyses were performed using IBM SPSS software, version 21 (IBM Corp., Armonk, NY).

## Results

Patient characteristics

The median age of the population was 60 years (interquartile range (IQR): 52-65). Ten of the 29 patients did not have any comorbidities. Diabetes was the most common comorbidity seen in 13 patients. Two patients developed MMMT after pelvic EBRT for squamous cell carcinoma of the cervix. One patient had previous breast cancer and a history of tamoxifen use. Bleeding per vagina was the most common presenting complaint seen in 62%, followed by abdominal pain in 55.2%. The mean age at menarche was 13 years (± 1.1). Except for one, all were post-menopausal. The mean age at menopause was 47.5 years (± 4.2). There was no nulliparity. Nearly 90% had more than two children. The median body mass index (BMI) was 23.3. About 22% of patients had a BMI greater than 25%. The mean baseline hemoglobin was 10.3 g/dl (± 1.2). 93% had a performance status of ≤2 (Table 1).

**Table 1:** Clinical characteristics of the patients BMI: body mass index; SD: standard deviation; IQR: interquartile range; n: number of patients

Demographic profile	
Age (years), median (range)	60 (IQR 52-65)
BMI, mean (±SD)	23.3 (±4.3)
Hemoglobin, mean (±SD)	10.3 (±1.2)
Postmenopausal, n (%)	28 (96%)
History of pelvic radiotherapy, n (%)	2 (7%)
Associated conditions	
Diabetes mellitus, n (%)	13 (44%)
Hypertension, n (%)	6 (20%)
Clinical features	
Vaginal bleeding, n (%)	18 (62%)
Lower abdominal pain, n (%)	16 (55.2%)
Mass per abdomen, n (%)	7 (24%)

Surgery and pathology

**Figure 1:**
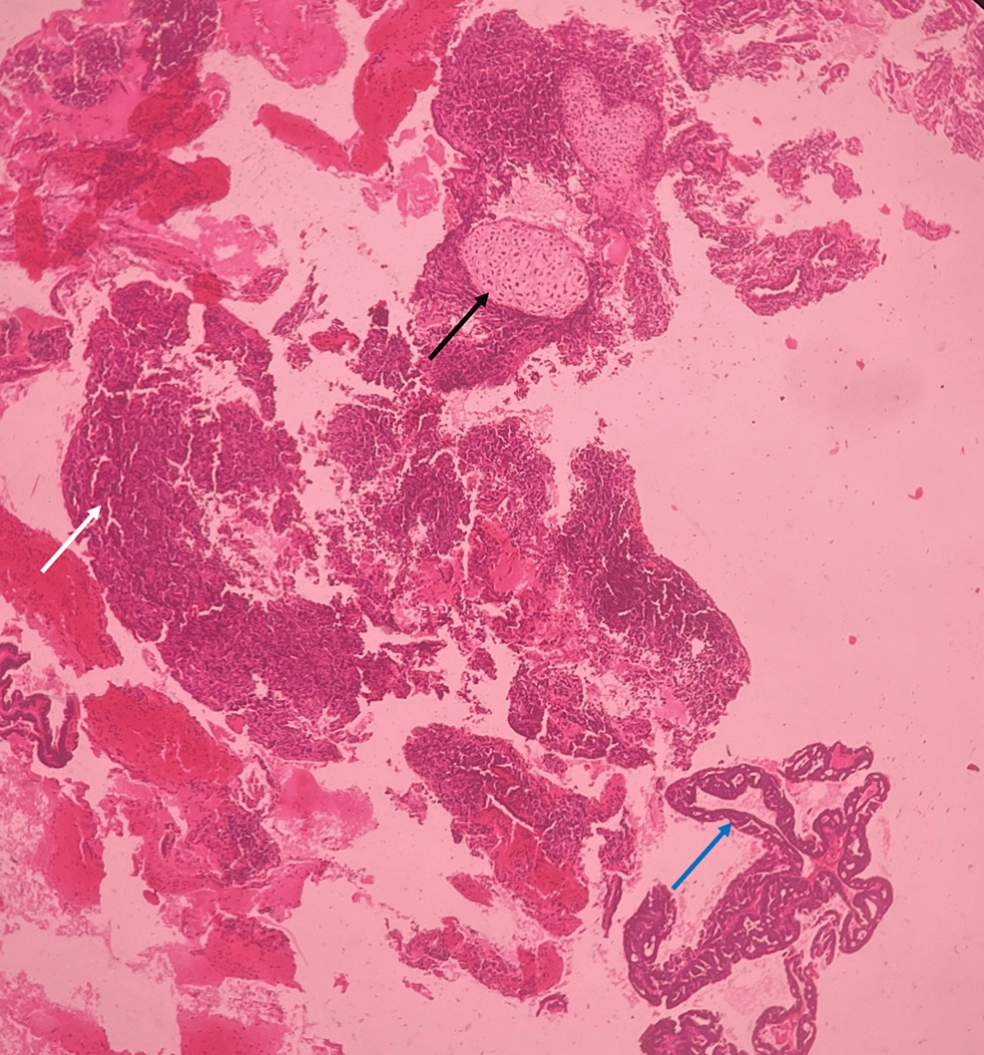
Histopathological examination of the uterine carcinosarcoma There is malignant biphasic cell proliferation composed of endometrioid-type histology in the carcinoma component (blue arrow), intimately admixed with sarcomatous elements like spindle cells (white arrow) and cartilage (black arrow).

The median size of the tumor was 7 cm (IQR: 6-10). Grades 2 and 3 comprised 89% of the population. Among the subtypes of MMMT, 21 were homologous, and the remaining eight were heterologous. The various heterologous elements seen were chondrosarcoma (five patients), osteosarcoma (one patient), angiosarcoma (one patient), and rhabdomyosarcoma (one patient). Figure 1 depicts the heterologous histology of a patient.

Two-thirds of the patients had tumors invading more than half of the uterine wall. Lymph vascular space invasion (LVSI) was seen in 42%. Cervical involvement was seen in 55% of the patients. Pelvic nodes were involved in five patients, and three had pelvic and para-aortic nodal involvement. Peritoneal cytology was positive in four patients (Table 2).

**Table 2:** Pathological characteristics of the patients FIGO: International Federation of Gynecology and Obstetrics; IQR: interquartile range; Nx: cancer in nearby lymph nodes cannot be measured; N0: no cancer in nearby lymph nodes; N1: the number and location of lymph nodes that contain cancer. The higher the number after the N, the more lymph nodes that contain cancer.

Pathological characteristics	Number of patients, n = 29
FIGO stage	
I	7
II	6
III	14
IV	2
Sites involved	
Cervix	3
Endometrium	22
Ovary	4
Grade	
1	3
2	11
3	15
Histopathology	
-Homologous	21
-Heterologous	
Rhabdomyosarcoma	1
Angiosarcoma	1
Bone	1
Cartilage	5
Myometrial involvement >50%	18
Positive peritoneal cytology	4
Lymph node status	
Nx	1
N0	22
N1	6
Size, median (range)	7.0 centimeters (IQR 6-10)

Adjuvant treatment

A total of four patients received neoadjuvant chemotherapy and subsequently underwent surgery. Around two-thirds of the patients received pelvic EBRT (Table 3).

**Table 3:** Treatment details

Treatment details	Number of patients, n = 29
Adjuvant chemotherapy	
Yes	17
No	12
Adjuvant radiotherapy	
Yes	19
No	10

Para-aortic nodal radiation was delivered to patients with positive para-nodal nodes in the operative specimen. Paclitaxel and carboplatin-based adjuvant chemotherapy were used in 60% of cases. As per institutional policy, adjuvant radiation was given in stages II and III, and adjuvant chemotherapy was given from stages II to IV. Some patients did not receive or complete the planned adjuvant radiation or chemotherapy due to intolerance, toxicities, and poor performance status.

Survival

The two-year disease-free survival (DFS) rate was 45%. The median DFS was 12 months (95% CI: 7.7-16.2). During the follow-up, 17 patients developed a recurrence (Table 4).

**Table 4:** Sites of recurrence

Site of recurrence	Number of patients	Treatment given
Local recurrence		
Isolated pelvic recurrence	2	Chemotherapy
Distant recurrence		
Intra-abdominal	9	Chemotherapy
Lung	4	Chemotherapy
Liver	2	Chemotherapy

**Figure 2:**
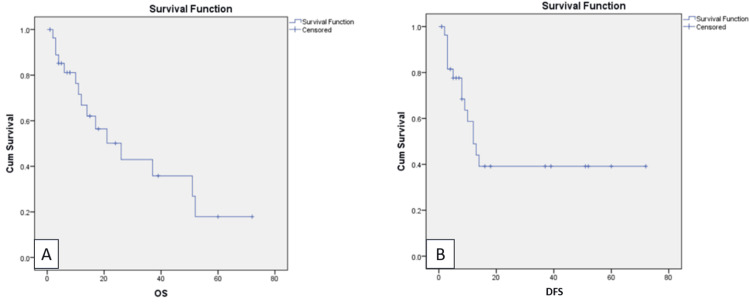
Kaplan-Meier estimates at 72 months A: overall survival; B: disease-free survival

Of the 17 patients, only two had isolated pelvic recurrence, and 15 developed distal recurrence. The most common site of distal recurrence was the peritoneal cavity, followed by the lung and liver. The commonly used second-line regimen was cisplatin/ifosfamide or single-agent ifosfamide based on the performance status. Single-agent pegylated liposomal doxorubicin was used as a third-line regimen. The median overall survival was 26 months (95% CI: 10.6-41.3). The three-year overall survival rate was 42% (Figure 2).

Prognostic factors

**Figure 3:**
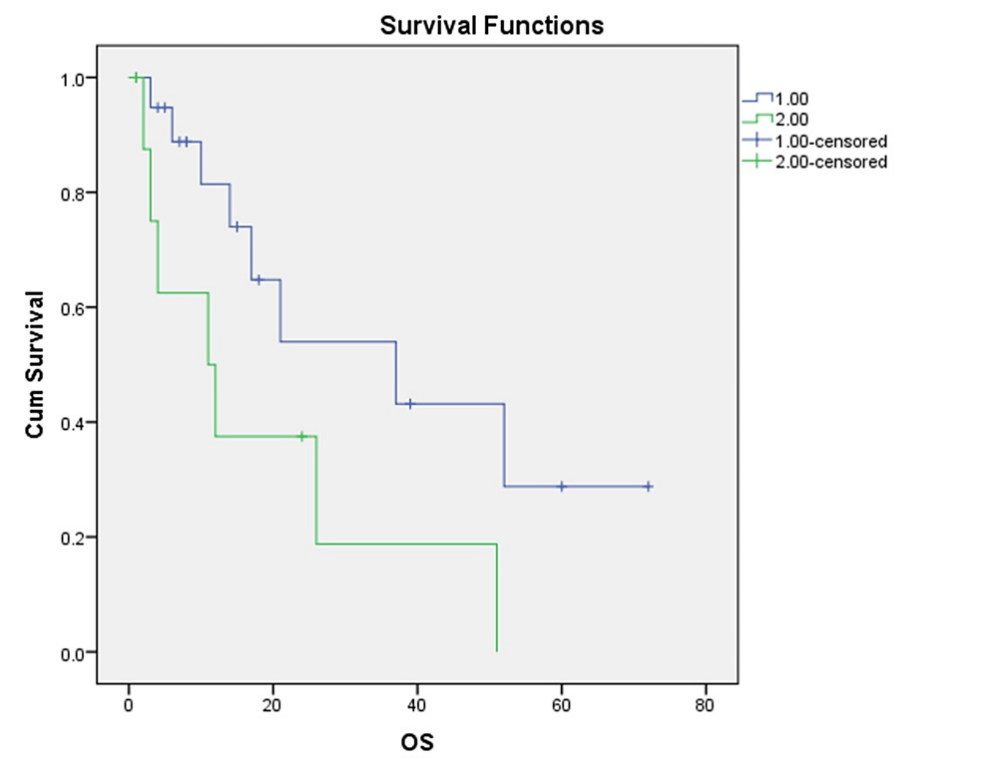
Addition of radiotherapy and outcome 1: with radiotherapy; 2: without radiotherapy

Univariate analyses were performed to identify prognostic factors. Patients with an age >60 years had a median overall survival of 11 months, compared to 37 months for those less than 60 years (p = 0.026). The median overall survival of patients with a tumor size >10 cm was 12.5 months, and it was 35 months when the tumor size was less than 10 cm (p = 0.03). Patients with a depth of myometrium invasion >half had poor survival, but this was not statistically significant (p = 0.079). Twenty-two percent of patients were obese with a BMI of more than 25 kg/m^2^. Patients’ LVSI, histological subtype, and cervical involvement were not found to be prognostic. Patients who had positive pelvic cytology had poor prognoses compared to those without it at 31 months vs. 16 months (p = 0.049). Patients receiving RT had an improved overall survival (39 months) compared to those who did not receive RT (12.5 months) ( p= 0.046) (Figure 3).

The addition of chemotherapy did not improve survival. There is no difference in survival between abdominal and non-abdominal recurrences. The patients who developed relapses had a median overall survival of 12 months. In the recurrence setting, the site of recurrence did not influence survival. Only four patients survived more than four years without evidence of recurrence.

## Discussion

Among uterine sarcomas, MMMT is the most common subtype [4]. Owing to the scarcity of data on MMMT in the Indian setting, we aim to present the clinical profile and outcomes of MMMT in our institute.

This is a retrospective study on the clinical profile and treatment outcomes of MMMT patients treated at our institute. Of the known risk factors associated with MMMT, in our study, 48% had diabetes mellitus, one patient had a history of tamoxifen usage, and two patients had exposure to radiation therapy [5-8]. The mean latency period between radiation exposure and cancer development was six years.

In Callister et al.'s study, post-menopausal status had an adverse influence on survival [3]. In our study, patients were mostly in the postmenopausal age group (96%), and they commonly present with vaginal bleeding. Also, 22% of patients were obese with a BMI of more than 25 kg/m^2^, and obesity has been observed as a risk factor in other studies as well [9, 10].

In our study, a tumor size of more than 10 cm was found to have a poor prognosis. We also found positive peritoneal cytology and depth of myometrium invasion to have a poor prognosis, as seen in other studies [2, 3].

Multimodal treatment is usually employed in the treatment of MMMT, owing to its aggressive course and poor prognosis [5, 11]. Surgical management usually includes total abdominal hysterectomy with bilateral salphingo-oophorectomy, infracolic omentectomy, and bilateral pelvic and para-aortic lymphadenectomy. The role of combined adjuvant chemotherapy and radiotherapy is still undefined [12]. Historically, ifosfamide alone was considered the most effective drug for MMMT, but now, based on the Gynecologic Oncology Group (GOG) 161 study, ifosfamide/paclitaxel is considered the standard of care [13, 14]. A recent GOG study by Powell et al. suggests that paclitaxel/carboplatin is equally effective as adjuvant chemotherapy [14, 15]. In our study, paclitaxel/carboplatin was used in the first-line adjuvant setting, and in subsequent therapy, an ifosfamide-based chemotherapy regimen was used, as recommended by the National Comprehensive Cancer Network (NCCN) guidelines.

Most of the patients received either chemotherapy or radiation therapy as a form of adjuvant therapy. In our study, patients receiving radiotherapy had an improved survival (39 months) compared to those who did not receive radiotherapy (12.5 months), p = 0.046 (Figure 3). The addition of chemotherapy did not improve survival. These findings of reduced local recurrence rates and improved overall survival with the addition of radiotherapy to chemotherapy when compared with chemotherapy alone were observed in other studies as well [11,16,17].

The median disease-free survival and overall survival were 12 and 26 months, respectively. As expected, the survival rates of our MMMT patients were poor, with a three-year overall survival rate of 42%. Among various factors, we found that elderly patients had poor prognoses, especially when their age was above 60 years, which has been reported in other studies as well [2, 18].

In the recurrence setting, the median overall survival was 12 months. Only four patients lived beyond four years without recurrence. As most patients were elderly with an ECOG performance status of two at baseline, only a few patients were fit for second-line chemotherapy. We did not observe any difference in survival between abdominal and non-abdominal recurrences.

The limitation of our study is that it is a retrospective study, which has its own bias. In our study, 70% underwent pelvic lymphadenectomy, but only 28% underwent para-aortic lymphadenectomy. Probably, better surgical staging is expected. Only 60% received adjuvant chemotherapy, and this number was very low in the recurrence setting owing to the poor performance status, thereby reducing survival.

## Conclusions

To conclude, MMMT has poor survival rates. Surgical resection remains the mainstay of treatment. Adjuvant radiation therapy improves survival. The age of the patient, tumor size, myometrium invasion, and positive peritoneal cytology were found to be prognostic factors. As it is a disease of the post-menopausal age group and predominantly seen in the elderly with comorbidities, performance status is one of the deciding factors for patients receiving subsequent therapy.
